# Ethical Attitudes of German Specialists in Reproductive Medicine and Legal Regulation of Preimplantation Sex Selection in Germany

**DOI:** 10.1371/journal.pone.0056390

**Published:** 2013-02-20

**Authors:** Miriam Wilhelm, Edgar Dahl, Henry Alexander, Elmar Brähler, Yve Stöbel-Richter

**Affiliations:** 1 Pediatrics 5 (Oncology, Hematology, Immunology; Gastroenterology, Rheumatology, General Pediatrics), Klinikum Stuttgart - Olgahospital, Stuttgart, Germany; 2 Institute for the Ethics of Medicine, University of Muenster, Muenster, Germany; 3 Department of Gynaecology and Obstetrics, University of Leipzig, Leipzig, Germany; 4 Department of Medical Psychology and Medical Sociology, University of Leipzig, Leipzig, Germany; University of Kansas Medical Center, United States of America

## Abstract

**Background:**

Because of its ethical and social implications, preimplantation sex selection is frequently the subject of debates.

**Methods:**

In 2006, we surveyed specialists in reproductive medicine in Germany using an anonymous questionnaire, including sociodemographic data and questions regarding ethical problems occurring in the practice of reproductive medicine. Most questions focused on preimplantation sex selection, including 10 case vignettes, since these enabled us to describe the most difficult and ethically controversial situations. This is the first survey among specialists in reproductive medicine regarding this topic in Germany.

**Results:**

114 specialists in reproductive medicine participated, 72 males (63%) and 42 females (37%), average age was 48 years (age range 29–67 years). The majority of respondents (79%) favoured a regulation that limits the use of preimplantation sex selection only for medical reasons, such as X-linked diseases (including 18%: summoning an ethics commission for every case). A minority of 18% approved of the use of sex selection for non-medical reasons (4% generally and further 14% for family balancing). 90% had received obvious requests from patients. The highest approval (46%) got the counselling guideline against a preimplantation sex selection and advising a normal pregnancy, if preimplantation sex selection would be allowed in Germany. The majority (67%) was opposed the personal use of preimplantation sex selection for non-medical reasons, but would think about it in medical cases. In opposite to woman, 14% of the men were in favour of personal use for non-medical reasons (p = 0,043). 25% of specialists in reproductive medicine feared that an allowance of preimplantation sex selection would cause a shift in the sex ratio.

**Conclusions:**

The majority of German specialists in reproductive medicine opposes preimplantation sex selection for non-medical reasons while recommending preimplantation sex selection for medical reasons, e.g. X-linked diseases like haemophilia.

## Introduction

Sex selection is a complex topic [1] with are a variety of social, economic, cultural and personal reasons for selecting sex of children [2]. The desire to choose the gender of one’s future child has probably existed since prehistoric times [3]. History teaches us that in large parts of the world pre-birth sex selection results in great discrimination against the birth of female offspring [4]. The fast changing landscape of reproductive technologies has experienced its share of controversies [5].

Sex can be selected via preconception and preimplantation sex selection, or by selective abortion [6].

Preconception sex selection offers the possibility of avoiding female infanticide [7]. MicroSort using Fluorescence in-situ hybridization (FISH) analysis of specimens pre- and post sort show that the 50∶50 X:Y ratio in unsorted spermatozoa can be shifted to 90% X or 75% Y after sorting [8]. However, MicroSort is less selective for children of the favoured sex than the preimplantation genetic diagnosis (PGD).

The PGD is a procedure that should not impair the development of the embryos. From its inception, PGD has been touted as an important alternative for couples who were carriers of autosomal recessive, dominant or sex-linked diseases [9].

As of today, 275 X-linked diseases or syndromes are known [10], [11]. Online Mendelian Inheritance in Man (OMIM, 2012) currently lists 1191 entries for genes and or phenotypes with X-linked inheritance. Most of the X-linked diseases affecting males only and vary in severity from colour blindness to haemophilia and Duchenne muscular dystrophy [12].

There are arguments that non-medical sex selection is the initial step down a road [13], eventually ushering in a world of designer children in which genetic engineering of offspring becomes routine [9].

### Law in Germany

According to §218a criminal code (StGB), while an abortion until the 12th week of pregnancy is not subject to prosecution in case of medical, criminological or psychological indication, it is definitaly prosecuted in case of sex-selective reasons. The new Genetic Diagnostic Law (GenDG) became applicable February 1, 2010 [14]. §15 forbid providing the information regarding the sex of the fetus or embryo to the parents before the end of the week 12 of pregnancy.

In Germany, preimplantation sex selection for non-medical reasons was forbidden by law [15]. At this time, PGD was not performed in Germany [16]–[18] because this would be an infringement against the embryo protection law ESchG [17].

Furthermore, the selection of sperm cells by using MicroSort is forbidden according to §3 ESchG, unless it helps avoid sex-linked genetic diseases [19].

A gynaecologist had examined eight extracorporeal fertilized oocytes at the stage of blastocyst and turned himself in to the court because of having performed a PGD. A recent judgement of the Federal Administrative Court (BGH) as of July 7, 2010, held that in Germany, a PGD is allowed in case of serious genetic defects [20]. After the debate of the German Bundestag, a new legal regulation was approved on July 7, 2011, which allowed PGD on its authorised centres after a positive vote of an ethics commission [21], indications are couples with serious genetic defects or in case of high risk of stillbirth or miscarriage. Non-medical sex selection for a future child remains still strictly forbidden. Previously PGD was not officially practiced in Germany, and the future application remains to be seen. After the new law regulation the first child using PGD was born on January 27, 2012, in the university Lübeck in Germany [22]. A legal decree from the Federal Ministry of Health was worked out and the German federal cabinet passed a legal decree for PGD on November 14, 2012. The legal decree must now be approved by the Federal Council of Germany [23].

Studies about the attitudes of the general population towards PGD are rare, even on the international level [24]. In Germany, there is considerable opposition to sex selection for non-medical reasons and to the selection of mental and physical characteristics of children [24].

## Methods

The results of this study emerge from the research project “Ethical attitudes of German specialists in reproductive medicine” by the Department of Medical Psychology and Medical Sociology of the University of Leipzig. Data was collected in a survey 2006 using a printed, sent by post, self-administered and anonymous questionnaire. A list of German specialists in reproductive medicine was compiled by the list of the German Society of Reproductive Medicine and the list of the German In Vitro Fertilisation (IVF) Registry. Out of 335 physicians asked to participate, 40.6% responded. The pretested questionnaire used to assess physicians attitudes towards preimplantation sex selection (by use of PID/MicroSort) contained questions about case vignettes, attitudes for and against sex selection, requests from patients, counselling directives, feared consequences, funding and regulation of sex selection. We examined measures of association between sociodemographic variables and answers to sex selection.

Data were analyzed with descriptive as well as inferential statistical procedures. Fisher’s exact test and χ^2^ analyses were used to compare unrelated probabilities, whichever appropriate. Continuous variables were compared by using Student’s t test. A two-sided p value of 0.05 or less was considered a statistically significant result. SPSS software, version 11.5, was used for statistical calculations.

Some questions are modified from a questionnaire from surveys among the German population [25]–[26] about sex selection or/and PGD. Furthermore, questions and case vignettes [27], used in the worldwide survey in 1985 and 1995 among human geneticists about pre-birth sex selection via prenatal diagnosis and consecutive abortion, were modified and added.

## Results

The subjects of this study were 114 specialists in reproductive medicine in Germany; there were 72 males (63.2%) and 42 females (36.8%). The average age was 48 years (age range 29–67 years). More information about the sample is summarized in Table 1.

**Table 1:** Sample.

Characteristic of the Sample	Men	Women	Total
N (%)	N = 72 (63.2)	N = 42 (36.8)	N = 114 (100)
**Age in Years**			
Average	50.3	45.0	48.3
Standard deviation	8.2	8.0	8.5
Range	33–67	29–67	29–67
Not specified	1 (1.4)	0	1 (0.9)
**Mostly Grown Up in**			
The newly-formed German states (former GDR)	10 (13.9)	12 (28.6)	22 (19.3)
The old West German states	59 (81.9)	27 (64.3)	86 (75.4)
Abroad	2 (2.8)	2 (4.8)	4 (3.5)
Not specified	1 (1.4)	1 (2.4)	2 (1.8)
**Family Status**			
Single	2 (2.8)	4 (9.5)	6 (5.3)
Married	61 (84.7)	32 (76.2)	93 (81.6)
Divorced	8 (11.1)	4 (9.5)	12 (10.5)
Widowed	1 (1.4)	2 (4.8)	3 (2.6)
**Children**			
Yes	63 (87.5)	29 (69.0)	92 (80.7)
No	8 (11.1)	13 (31.0)	21 (18.4)
Not specified	1 (1.4)	0	1 (0.9)
**Highest Academic Qualification**			
Diploma	1 (1.4)	2 (4.8)	3 (2.6)
Conferral of a doctorate	45 (62.5)	34 (81.0)	79 (69.3)
Habilitation	23 (31.9)	2 (4.8)	25 (21.9)
None of these	3 (4.2)	4 (9.5)	7 (6.1)
**Religion**			
Catholic	21 (29.2)	7 (16.7)	28 (24.6)
Evangelic	23 (31.9)	17 (40.5)	40 (35.1)
Other religion	2 (2.8)	3 (7.1)	5 (4.4)
No religion	24 (33.3)	15 (35.7)	39 (34.2)
Not specified	2 (2.8)	0	2 (1.8)
**Religiousness**			
1 (not religious)	20 (27.8)	14 (33.3)	34 (29.8)
2	16 (22.2)	7 (16.7)	23 (20.2)
3	20 (27.8)	12 (28.6)	32 (28.1)
4	9 (12.5)	7 (16.7)	16 (14.0)
5 (religious)	5 (6.9)	1 (2.4)	6 (5.3)
Not specified	2 (2.8)	1 (2.4)	3 (2.6)

### Case Vignettes

**Figure 1:**
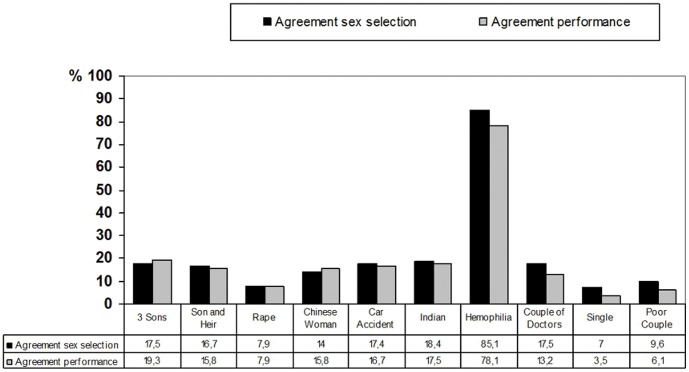
Case Vignettes. Agreement to preimplantation sex selection for special cases in Germany (left beam) und agreement to self-performance of preimplantation sex selection (right beam) in these cases, if it were allowed in Germany.

The usage of case vignettes (Table 2; Figure 1) allowed us to describe the most difficult and ethically controversial situations that may challenge a respondent’s general opinion.

**Table 2:** Case Vignettes.

**(1) 3 Sons:**	One couple, which already has three sons, wishes to have a daughter. Already during her second and third pregnancy, the wife explains, she hoped that it might be a girl this time. Unfortunately, it didn’t work that way. She says: “Please don’t get me wrong, I really love my sons wholeheartedly. But still, I simply want to know what it is like to have a daughter. As I know it in the relationship to my own mother, a mother-daughter relationship is somehow deeper and emotionally more satisfying than a mother-son relationship.”
**(2) Son and Heir:**	A Couple that already has two adult daughters wants a son. As the husband explains, the older daughter became a nurse and the younger daughter became a kindergartner. He wishes to have a son who could take over the garage that he himself inherited from his own father and which he is deeply committed to.
**(3) Rape:**	A single woman is desperate to have a daughter. As she entrusts to the doctor, she says she was the victim of rape at the age of 16. Since then, she says she has lost all confidence towards men. Although she can’t think of getting married at any time, she still wants to have her own child. As she believes that she would be a far better mother to a daughter than to a son, she would like to have a daughter.
**(4) Chinese Woman:**	A woman who was born in China and who already has two daughters insists on a son. She says that her husband has threatened to send her and her daughter to the mainland if she doesn’t give him a son. She is so distressed that she pronounces: “If you don’t help me, I will take a test after my next pregnancy that will tell me the gender of the baby. In the case of another daughter, I will undergo an abortion.”
**(5) Car Accident:**	The parents of four boys and one girl have lost their only daughter in a car accident. They wish to have a new girl. They say: “We know, we can’t replace our daughter. But we are convinced that the birth of a girl could heal the scars that the sudden death of our only daughter has left. Since we have heard of the possibilities of the prebirth sex selection, we look into the future more than into the past.”
**(6) Indian:**	A couple from India that already has two daughters wants a son. The husband is deeply religious. He explains that according to Hindu beliefs, a man will only go to heaven if he leaves a son who performs the sacrifice of the death and who continues the cult of the ancestor spirits. Those, however, who neglect to father a son, will cross with the ancestors and must go to hell.
**(7) Hemophilia:**	A couple is desperate for a daughter. The father of the woman is suffering from hemophilia A, which is transferred to offspring through an X-linked recessive hereditary disease. The woman is free of complaints, but a genetic test showed that she is a heterozygous carrier. The man is healthy concerning this. A son would have a 50 percent probability of suffering from haemophilia. A daughter would have a 50 percent possibility of being an asymptomatic carrier of the disease. Being informed of the severity of the disease, they wish to have a healthy child and therefore a daughter.
**(8) Couple of Doctors:**	A couple of doctors at the age of 40 have two teenage sons. They could imagine raising another child, assuming it would be a girl. They want to use a PID for sex selection and for exclusion of chromosomal disorders like trisomy 21 because of their age.
**(9) Single:**	A woman wants a girl. She believes that girls are easier to rear. As she would be a single mother, this is very important for her.
**(10) Poor Couple:**	A couple that possesses only a low income asks you for help: “We want a son very much, and our funds are very limited. We can only afford to have one child, and we want to provide it a more or less normal life. If we would leave that to chance and simply give birth to several children in order to get a son, we would get into financial troubles and would have little to offer to our children.”

Respondents were asked whether preimplantation sex selection should be allowed in Germany and if they would perform a sex selection in this case, if it would be allowed in Germany. For non-medical cases, the majority is against allowing preimplantation sex selection in Germany, and they are also not willing to perform preimplantation sex selection (Figure 1).

The case vignette 7: Haemophilia - as a medical indication gets agreement from the majority of respondents. 85.1% endorse this case to be allowed in Germany, and 78.1% would be willing to perform preimplantation sex selection. In the case vignette 9: Single - only 3.5% of medical specialists in reproductive medicine would perform a preimplantation sex selection.

In seven of ten case vignettes, there is a significant difference between the genders (p<.05). Men are more willing to perform a preimplantation sex selection than women. On average, women are five years younger than men and worked shorter than male specialists in reproductive medicine in general (women median was 16.6 years, men 22 years) as well as in the field of reproductive medicine (women median was 11.3 years, men 15.3 years). The Fisher’s exact test regarding the legal regulation of preimplantation sex selection in Germany shows no significant difference regarding the formation of groups: Age (p = .749), man-years as physician in general (p = .506) and in the field of reproductive medicine (p = .922) as well as single factor variance analysis regarding age and working experience (SNK p>.05).

### Prenatal Diagnostics (PND)

A case vignette deals with a couple aged 27 requesting prenatal diagnostic (PND). The reason given for this request is that they have had a child with trisomy 21 who died, but they have no documents to prove this. After being tested, they show an unusual interest in learning the child´s sex.

The majority (78.9%) of respondents would ask the couple whether it would use that test for a pre-birth sex selection (Table 3). 73.7% did already have such cases. 66.7% would not tell the gender within the legal time for abortion. By doing so, the majority of specialists in reproductive medicine feels that they have moral commitments by taking their right of knowledge transfer, which is opposed to the right of knowledge of the patients. This is in contrast to the claim that 96% of the patients advocate that parents have the right to find out the sex of the child [28].

**Table 3:** Case Vignette Prenatal Diagnostics (PND).

Prenatal Diagnostics	Not specified	N (%)	Yes	N (%)	No	N (%)
I would ask the couple whether it uses the test for sex selectionF = 2,37 p = 0,316	6	(5.3)	90	(78.9)	18	(15.8)
I would inform the parents about the gender within the periodof a legal abortion.χ^2^ = 2,83 p = 0,259	14	(12.3)	24	(21.1)	76	(66.7)
I have already had cases in which I had the suspicion that thereason for the prenatal diagnosis was in reality a sex selectionχ^2^ = 0,75 p = 0,811	15	(13.2)	15	(13.2)	84	(73.7)

A couple aged 27 requests prenatal diagnosis. They say that they had a child with Down syndrome who died, but they have no documents to prove this. After you have performed the test, they show an unusual interest in learning the child´s sex.

### Legal Regulation of Preimplantation Sex Selection in Germany

Respondents were asked for what solution our society should choose regarding the regulation of new reproductive technologies for sex selection (Table 4). The majority of respondents (78.9%) is in favour of a regulation that limits the use of preimplantation sex selection only for medical reasons, such as X-linked diseases (including 17.5% summoning an ethics commission for every case). A minority of 18.4% approve of the use of sex selection for non-medical reasons (4.4% generally and a further 14% for family balancing). The answers show no significant gender-specific differences (p = .213).

**Table 4:** Possibilities of sex selection regulation in Germany.

Reproduction techniques for preimplantation sex selection should be:	N	(%)
Possible for everyone without restriction	5	(4.4)
Available for couples with gender-linked diseases or with 2 or 3 children of the same genderwho wish a child of the other sex	16	(14.0)
Only made available to couples with sex-linked diseases	70	(61.4)
After an in-depth evaluation and permission from a special committee (ethics commissiondecides every case separately)	20	(17.5)
Banned by the German Medical Association, infringement by doctors should be punishedwith reprimand, fine or in severe cases withdrawal of approval	0	
Prohibited under criminal law, infringement should be prosecuted with imprisonmentup to one year or incur a heavy financial penalty	2	(1.8)
No answer	1	(0.9)

### Counselling Guideline

Respondents were asked about their counselling guideline, if preimplantation sex selection were allowed in Germany (Table 5). The highest approval (45.6%) goes to the counselling guideline against a preimplantation sex selection and advising a normal pregnancy, if preimplantation sex selection were allowed in Germany. 14.9% would not only be willing to counsel but also to perform a preimplantation sex selection.

**Table 5:** Counseling Directive.

Counseling Directive	N	(%)
No answer	1	(0.9)
I would dissuade the parents from a preimplantation sex selection and rather recommend a “normal“pregnancy.	52	(45.6)
I would recommend a normal pregnancy with the option to put the child up to adoption if it doesn’t have the preferred gender.	1	(0.9)
I would give advice to the parents concerning reproduction techniques (only provide information).	33	(28.9)
In the case of a decision in favor of a preimplantation sex selection, I would not only give advice to the parents,but also support them by recommending them to a colleague.	8	(7.0)
In the case of a decision in favor of a preimplantation sex selection, I would not only give advice to the parents,but also support them by carrying out the operation myself.	17	(14.9)
Something else	2	(1.8)

There is a significant difference regarding counselling guidelines between genders (p = .020). Men would much more frequently not only be willing to counsel but also to perform a preimplantation sex selection (22.2%; women: 2.4%). Women would more often dissuade from preimplantation sex selection and recommend a normal pregnancy (52.4%; men: 41.7%) or they would only counsel (35.7%; men: 25%).

25.4% of specialists in reproductive medicine fear that allowing preimplantation sex selection would cause a shift in the sex ratio. 14% worry that more boys would be born. A shift towards girls is not expected.

## Discussion

After the debates and the new law regulation, PGD for sex selection caused heated debates among the German population and specialists. This is the first study focused on this topic and the ethical attitudes of German specialists of reproductive medicine. Even if this study was done 2006, there are no new studies published till now.

The majority (67%) of German specialists of reproductive medicine would not tell the gender within the legal time for abortion, according to the guidelines from the society and the new law regulation [14]. In the United States obstetrician-gynaecologists are more in favour of sex selection. In a survey in the year 2008/2009 among 1,154 U.S. obstetrician-gynaecologists 64% would help the patient to obtain an abortion for sex selection [29]. The majority of units in Finland made fetal sex determination during the second-trimester ultrasonographic screening without medical indication at patient`s request [30].

While in 1995 [27], [31] less than a half of the interviewed German human geneticists (47%) had received obvious requests of sex selection, 90% indicate that they have had such requests in the current survey.

In 1995, 90% of German human geneticists were opposed to pre-birth sex selection for non-medical reasons (asked in case of abortion), while in 1985 it was still 98% [27], [31]. The majority of the population and the majority of specialists in reproductive medicine today are still opposed to preimplantation sex selection for non-medical cases.

The Human Fertilisation and Embryology Authority (HFEA, dedicated to licensing and monitoring UK fertility clinics and all UK research involving human embryos) stated, that centres should not select the sex of embryos for social reasons and centres should not use sorting techniques in sex selection [32]. The European Society of Human Reproduction and Embryology (ESHRE) as the European body for professionals in reproductive medicine and biology did not strictly ban non-medical sex selection - which is not allowed in EU, but after an ethical debate they report these data [33].

As a representative sample of the German population in 2003 [25], 1005 men and women 18 years and older were asked whether preimplantation sex selection should be made available or not. 32% held that sex selection should be strictly prohibited, be it for medical or non-medical reasons, compared to only 2% of specialists in reproductive medicine in 2006. The tendency to agree to a restrictive legalisation for PGD in Germany can be observed in the majority of respondents. 54% accepted the use of preimplantation sex selection for medical purposes (specialists in reproductive medicine 2006 were 61% and another 18% for summoning an ethics commission). Only a minority of 11% approved the use of sex selection for non-medical reasons (specialists in reproductive medicine 2006 - 4% approve it generally and further 14% approve it for couples with at least two children of the same gender and who wish a child of the different gender). Specialists in reproductive medicine use techniques of reproductive medicine for other medical indications; perhaps there is less inhibition in comparison to the general population to agree on the use of techniques of reproductive medicine even for X-linked diseases as a medical indication. It might be that specialists in reproductive medicine are more tolerant than the general population, precisely because they are confronted with the individual fates of patients on a daily basis.

A survey among the general population realized in 2004 [24] resulted in a stronger disaffirmation of preimplantation sex selection for 92% of the sample. Only a minority advocates the preimplantation sex selection (8%) and would personally make use of it (6%), while the majority is for a restricted permission of PGD in Germany. 76% plead for allowing PGD in case of a disease which may cause death within the first year of life. 63% consider using the method for themselves in such a case.

In a web poll in 2007 targeting infertile people with a wish for a child [34], the disaffirmation of preimplantation sex selection compared to the survey from 2004 was lower (83%, in 2004∶92%), if the techniques used would require several treatment cycles and corresponding costs for the couple and only 13% considered using the technique if the costs were to be covered by a health insurance company and limited to one treatment cycle.

43% of specialists in reproductive medicine demand the exclusive treatment of couples as criterion and reject the treatment of singles. This is reflected in the case vignettes, in which the treatment of singles is more often refused than the treatment of couples.

Between July 2003 and July 2005, a cross-sectional study [35] was carried out with a sample of health care professionals working in assisted reproductive medicine in Brazil, Germany, Greece and Italy. 224 persons participated in the study, of these 50 Germans (39 with medical profession). One of the cases described a case of non-medical sex selection: “A heterosexual couple, who has two children, goes to a Human Reproduction Centre because they whish to have another child, yet due to a tubal problem, she is unable to have her ovum fertilized naturally. Considering their request involves a technical procedure and they already have two boys, they would only like female embryos to be implanted.” 76% would perform the procedure and 24% would not (no separate results for German professionals provided). In opposite to the current study under German specialists in reproductive medicine, more professionals from the international study are in favour of performing a sex selection for non-medical reasons.

In the survey among human geneticists [27], [31] it was possible to write answers in a free text field for reasons for positive and negative answers towards the case vignettes using prenatal diagnosis and consecutive abortion in cases of unpreferred gender (Table 6 “worldwide” – no separated German data available), for to examine personal attitudes and moral values. In the present analysis however, there was the possibility to agree with one of the predefined answers regarding preimplantation sex selection (PGD/MicroSort) and optionally insert another answer in a free text field.

**Table 6:** Reasons of Approval or Rejection in at least one of the Case Vignettes.

	Human geneticists“worldwide” 1995 (sexselection via sex-selectiveabortion)	Specialists in reproductive medicineGermany 2006 (sex selection via PID/MicroSort)
**Reasons of Approval**		
1. In deference to the autonomy of the patient	29%	15.8%
2. In deference to the culture or the religion of the patient	6%	13.2%
3. Retain the unity of the family	3%	6.1%
4. The patients have the right of any service they can pay for	8%	0
**Reasons of Rejection**		
1. Prevent a possible gender discrimination	7%	71.9%
2. Prevent the misuse of reproduction techniques which areintended to identify genetically caused diseases	43%	51.8%
3. The refusal of the abortion of a healthy embryo	16%	29.8%
4. The right of a professional to refuse a service	7%	34.2%
5. To avoid the tendency of a cosmetic selection	1%	50.0%
6. To preserve a balance of genders	3%	21.9%

29% of the human geneticists recommend the autonomy of the patients (specialists in reproductive medicine 16%) and 8% agree that the patients have the right to obtain any service they can pay for (specialists in reproductive medicine 0%). For specialists in reproductive medicine today, other ethical values are in the foreground, e.g. the right of the expert to refuse a service got an approval of 34% (human geneticists 7%). Otherwise, a refusal of sex selection in order to avoid gender discrimination got an approval of 72% of the specialists in reproductive medicine (human geneticists: 7%).

For specialists in reproductive medicine, the responsibility of the physician is more focused, i.e. a physician is acting concerning his ethical values, while human geneticists in the survey from 1995 understood themselves more as service providers and were thereby more focused on the patient’s rights and the patient’s self-determination.

China and India reveal a significant son preference [36]. Girls were abandoned, neglected or even killed after birth [37] and due to the discrimination of female descendants, there is a risk of strengthening sexual stereotypes [1], [4].

In Europe and the USA, a preference for the male gender was found in two-thirds of the cycles of non-medical preimplantation sex selection [37], [38]. In India and China, there is a preference for boys. Therefore, female descendants are discriminated, and there is the danger of a boost of gender stereotypes [1], [4]. With the use of sex-selective abortion, which became available in the mid-1980s, there are now an estimated 80 million missing females in India and China alone [39]. A worldwide allowance of preimplantation sex selection would increase this imbalance.

In 2007 [34] in Germany, the preference for girls (19%) was higher than the preference for boys (11%). In an older survey from 2005 [37], 14% wanted to have a boy while 10% wanted a girl as their first-born child. In Germany, a shift in the sex ratio caused by allowing preimplantation sex selection is not likely, because among the population there is little interest in the use of preimplantation sex selection on the one hand, and there is no significant gender preference on the other hand.

The use of reproductive techniques to fulfil the desire to have children is minimal. Only 1.65% of children are born with the help of IVF in Germany [40]. In Germany 2010 from 50,583 oocyte retrievals led to an IVF or ICSI treatment, 1,074 children were born after IVF, 3,856 after ICSI and 108 after IVF/ICSI [41]. Preimplantation sex selection offers an opportunity for patients with X-linked disease to get a healthy child, which is free of this disease. Fragile X syndrome was the most common indication followed by Duchenne muscular dystrophy and haemophilia [33].

The risk of birth defects associated with ICSI, but not IVF, remained increased [42]. Robert G. Edwards did win 2010 the Nobel Prize for the development of IVF [43]. This year`s Nobel Prize winners John Gurdon [44] established the fundamental principles for Shinya Yamanka sensational discovery to induct pluripotent stem cells from mouse embryonic and adult fibroblast cultures [45]. The reconstruction of female germ-cell development in vitro is a key challenge in reproductive biology and medicine. A team from Kyoto University used stem-cell technology to create viable egg cells in laboratory mice that were fertilised by IVF to produce normal, healthy offspring [46]. But with more techniques available, more ethical controversy debates will raise. Today, the prenatal detection of Down syndrome or also Edwards syndrome is with a simple blood test available, used cell-free DNA in the blood of the pregnant woman [47]–[48].

In the case of a general allowance of preimplantation sex selection, there is a fear of a trend towards designer-babies and even towards eugenics [49]. Parents could be willing to choose other characteristics [50], e.g. intelligence or the colour of the eyes. Should the autonomy of the patient be possible without restriction and is a selection of medically irrelevant characteristics permissible [51]? Regarding PGD, it is necessary to scrutinize from which point in time human life is defined and whether an embryo is to be seen as a living being with the status of a person [52]. Furthermore, the application of PGD is showing new ethical problems, e.g. there are surplus cryoconserved embryos which then partly have to be rejected [53].

The paper was sent out to the members to the German Society of Reproductive medicine and to German IVF centres. It is possible that the members of the Society did give an individual answer and that the answer of the same person is given by the IVF centre under the name of the centre. It is not completely excluded that double answers were given. In fact it cannot be ruled out that differences in answers are possible for nonrespondents with a response rate of 40.6%. These are the limitations of the study.

This German survey from 2006 showed the willingness of German doctors of reproductive medicine to perform a preimplantation sex selection only for medical reasons. Because of the German history and because PGD for non-medical reasons remains still forbidden today, this attitude will not have mainly changed. There is a high moral principle in Germany regarding the need for protection and selection of human life and the wrong gender itself is not a sufficient reason. This is the first study focused on this topic preimplantation sex selection and the ethical attitudes of German specialists of reproductive medicine. Even if this study was done 2006, there are no new studies published till now. Limitation of this study is that the new law regulation and the upcoming debates may have an effect on attitudes and further studies are necessary to evaluate this.

### Conclusion

The majority of German specialists in reproductive medicine is against preimplantation sex selection for non-medical reasons, while they recommend the permission of preimplantation sex selection for medical reasons, for example X-linked diseases like haemophilia.

Obviously, sociodemographic variables did not play an essential role regarding the attitude of German specialists in reproductive medicine. Instead, personal attitudes and moral values have an effect.

This German survey from 2006 showed the willingness of German doctors of reproductive medicine to perform a preimplantation sex selection only for medical reasons. This attitude will not have mainly changed. Since the judgement of the Federal Administrative Court (BGH) as of July 2010 and the new law regulation in July 2011, the PGD is allowed in case of serious genetic defects. PGD for non-medical reasons remains still forbidden. Previously, PGD was not officially practiced in Germany, and the future application remains to be seen. If it were allowed without restrictions, preimplantation sex selection would still only be used in individual cases in Germany. To find law regulation for every individual case would seem justified, but is not realistic. In this context, every case should be discussed in an ethics commission.
